# Delayed Cardiac Tamponade Caused by Right Atrial Perforation from a Fractured Permanent Inferior Vena Cava Filter: A Case Report

**DOI:** 10.70352/scrj.cr.26-0047

**Published:** 2026-06-06

**Authors:** Kensuke Oue, Moe Kinoshita, Nobuo Kondo

**Affiliations:** Department of Cardiovascular Surgery, Kochi Health Sciences Center, Kochi, Kochi, Japan

**Keywords:** inferior vena cava filter fracture, cardiac tamponade, right atrial perforation, permanent inferior vena cava filter, delayed complication

## Abstract

**INTRODUCTION:**

Inferior vena cava (IVC) filters are used to prevent pulmonary embolism in patients with deep vein thrombosis (DVT). Although generally safe, long-term indwelling filters may cause complications such as fracture, migration, and organ perforation. Cardiac tamponade due to right atrial (RA) perforation is extremely rare, particularly with permanent filters.

**CASE PRESENTATION:**

A 60-year-old woman presented to our institution with sudden epigastric pain. She had undergone permanent IVC filter placement 13 years earlier for DVT and had remained on warfarin thereafter. At the initial hospitalization, electrocardiography (ECG) showed mild ST-segment depression and cardiac biomarkers were slightly elevated. Transthoracic echocardiography and contrast-enhanced CT showed no pericardial effusion, and coronary angiography (CAG) revealed no significant coronary stenosis, although a linear radiopaque structure was incidentally observed near the cardiac silhouette. Vasospastic angina was suspected, and she was discharged after symptom improvement. Three days later, she returned with worsening dyspnea and general fatigue. Transthoracic echocardiography revealed pericardial effusion, and contrast-enhanced CT demonstrated a hyperdense fragment in the RA, additional fragments around the right ventricle (RV), and penetration of the filter into the duodenum. Comparison with CT obtained 4 years earlier showed a newly developed structural defect of the filter, consistent with fracture. Warfarin was discontinued, and no heparin bridging was performed because of the bleeding risk. The patient underwent a sequential open surgical approach consisting of laparotomy followed by median sternotomy, with filter removal and repair of the duodenal perforation performed during the abdominal phase and removal of the migrated fragment and repair of the RA perforation performed under cardiopulmonary bypass during the cardiac phase. Her postoperative course was uneventful, with no recurrent pericardial effusion or IVC obstruction during 2 years of follow-up.

**CONCLUSIONS:**

Late structural failure may occur even in permanent IVC filters, and migrated filter components can cause life-threatening cardiac tamponade long after implantation.

## Abbreviations

CAG
: coronary angiography
CK
: creatine kinase
CK-MB
: creatine kinase-MB
CRP
: C-reactive protein
DVT
: deep vein thrombosis
ECG
: electrocardiography
IVC
: inferior vena cava
PT-INR
: prothrombin time-international normalized ratio
RA
: right atrium
RV
: right ventricle

## INTRODUCTION

IVC filters are used to prevent pulmonary embolism in patients with DVT or contraindications to anticoagulation. Long-term indwelling filters may cause complications such as penetration, fracture, and migration, although life-threatening cardiac events are rare.^1–5)^ We report a rare case of delayed cardiac tamponade caused by RA perforation from a fractured permanent IVC filter 13 years after implantation.

## CASE PRESENTATION

**Fig. 1:**
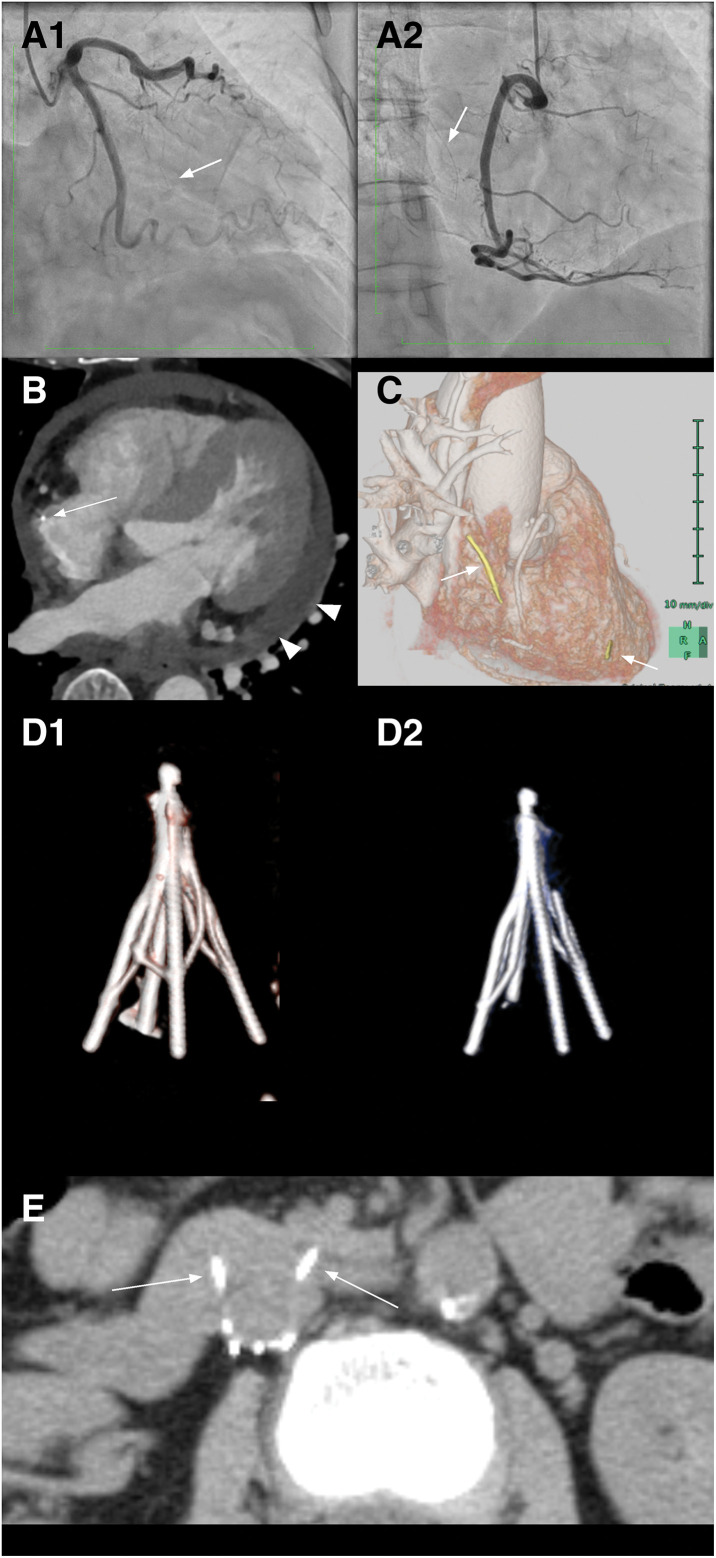
Preoperative imaging findings. (**A**) CAG showing a linear radiopaque structure near the cardiac silhouette (arrows). (**A1**) Left CAG. (**A2**) Right CAG. (**B**) Contrast-enhanced CT showing pericardial effusion (arrowheads) and a hyperdense structure within the RA (arrow). (**C**) 3D CT reconstruction demonstrating hyperdense fragments located in the RA and around the RV, showing a strut-like morphology. (**D**) 3D CT comparison of the IVC filter. (**D1**) 3D image obtained 4 years earlier demonstrating an intact IVC filter configuration. (**D2**) Current 3D image demonstrating a defect in the filter structure, consistent with filter fracture. (**E**) Contrast-enhanced CT showing penetration of the IVC filter through the IVC wall into the duodenum (arrow). CAG, coronary angiography; IVC, inferior vena cava; RA, right atrium; RV, right ventricle

A 60-year-old woman presented to our institution with sudden epigastric pain on December 24. She had undergone permanent IVC filter placement 13 years earlier for DVT. Her medical history included hypertension, and she had remained on warfarin following permanent IVC filter placement. On admission, ECG showed mild ST-segment depression. Laboratory tests showed hemoglobin 14.1 g/dL, white blood cell count 6760/μL, CRP 0.19 mg/dL, troponin I 172.4 ng/mL, CK 184 U/L, CK-MB 23.3 U/L, PT-INR 1.78, and serum creatinine 0.57 mg/dL. Transthoracic echocardiography showed no regional wall motion abnormality or pericardial effusion, and contrast-enhanced CT also demonstrated no pericardial effusion. Because unstable angina was initially suspected, CAG was performed. CAG revealed no significant coronary stenosis; however, a linear radiopaque structure was incidentally observed near the cardiac silhouette (**Fig. 1A**). Its clinical significance was unclear at that time, and it was not considered to be the cause of the patient’s symptoms. Vasospastic angina was therefore considered, and she was discharged the following day after symptom improvement. Three days later, she returned to our institution because of worsening dyspnea and general fatigue. At the second presentation, hemoglobin was 11.8 g/dL, white blood cell count 7300/μL, CRP level 5.22 mg/dL, troponin I 19.8 ng/mL, CK 58 U/L, CK-MB 15.2 U/L, PT-INR 1.89, and serum creatinine 0.55 mg/dL. Transthoracic echocardiography performed on arrival revealed pericardial effusion, raising suspicion of cardiac tamponade. Contrast-enhanced CT demonstrated a hyperdense structure within the RA (**Fig. 1B**).

3D CT reconstruction allowed visualization of hyperdense fragments located in the RA and around the RV. These fragments exhibited a strut-like morphology (**Fig. 1C**). 3D CT reconstruction of the IVC filter demonstrated an apparent structural defect compared with 3D imaging obtained 4 years earlier, which had shown an intact filter configuration (**Fig. 1D**). This temporal comparison provided direct evidence of filter fracture. CT also demonstrated penetration of the IVC filter through the caval wall into the duodenum (**Fig. 1E**), and retrospective review of the prior CT showed that this finding had already been present 4 years earlier. Once the diagnosis of cardiac tamponade caused by a fractured filter wire was established, warfarin was discontinued. Heparin bridging was not performed because of the active bleeding risk, and surgery was undertaken on the following day.

### Surgical findings

#### Abdominal phase

**Fig. 2:**
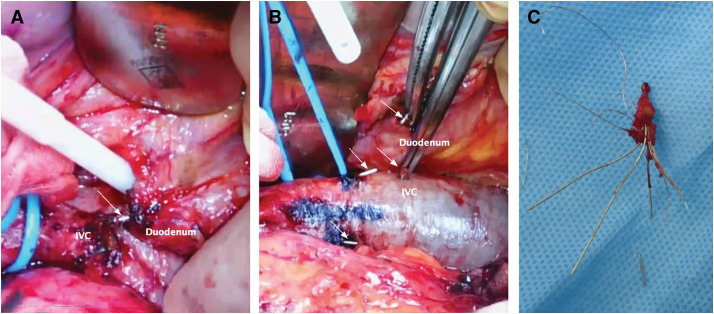
Operative findings during the abdominal phase and retrieved IVC filter. (**A**) Intraoperative photograph demonstrating the anatomical relationship between the IVC and the duodenum. One wire component of the IVC filter is seen penetrating from the IVC into the duodenum (arrow). (**B**) Intraoperative photograph after division of the 2 penetrating wires between the IVC and the duodenum. Arrows indicate the divided wire ends. (**C**) Retrieved IVC filter specimen showing disruption and loss of a wire component connecting the filter struts. IVC, inferior vena cava

Laparotomy was performed through an upper midline incision. After mobilization of the duodenum, the IVC was exposed, and a filter leg was found to penetrate extravascularly through the right lateral wall, while the left side of the IVC was firmly adherent to the duodenum. The IVC was taped at the suprarenal and infrarenal levels and at the left renal vein confluence; all tapes were snared, and a longitudinal cavotomy was performed. The filter was removed under direct visualization of the lumen while carefully dissecting the white neointimal tissue covering the filter body and struts. The wire penetrating the duodenum was also removed (**Fig. 2A** and **2B**), and the duodenal perforation was closed with interrupted 4-0 polydioxanone sutures. Because the posterior wall of the IVC at the level of the left renal vein became deficient after removal, it was repaired from inside the lumen with 5-0 polypropylene sutures. One wire component running from the anterior caval wall toward the vertebral body was intentionally left in place because it was completely covered by neointima and could not be removed without resecting the caval wall.

Examination of the retrieved filter specimen revealed disruption and loss of a wire component connecting the filter struts, while the struts themselves remained intact (**Fig. 2C**). No systemic heparinization was used during the abdominal phase because of the risk of bleeding. After removal of the IVC filter and repair of the duodenal perforation, the abdominal field was temporarily packed and covered. Final abdominal closure was performed after completion of the cardiac procedure and reversal of heparin with protamine, allowing reconfirmation of hemostasis under normalized coagulation status.

#### Cardiac phase

Median sternotomy was subsequently performed. After the pericardium was opened in an inverted T-shaped fashion and a large amount of dark red bloody pericardial effusion was encountered, systemic heparinization was initiated, and cardiopulmonary bypass was established once the activated clotting time exceeded 400 s. A clot was identified in the right atrioventricular groove, and after its removal, a wire fragment was found to be protruding beyond the outer surface of the RA. Along the extension of the fragment, the pericardium was deeply eroded toward the right pleural cavity and reached the lung parenchyma, a finding considered consistent with repeated mechanical friction between the fragment and the pericardium. The opposite end of the fragment had produced a subepicardial hematoma on the right atrial lateral wall, but it had not penetrated into the pericardial cavity.

**Fig. 3:**
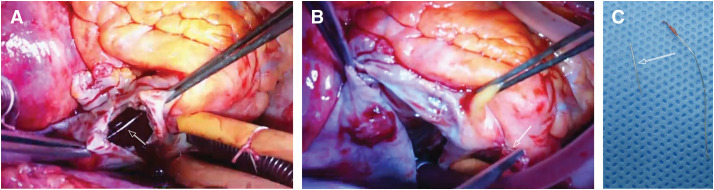
Operative findings during the cardiac phase. (**A**) Intraoperative view after right atriotomy showing a wire-like metallic structure within the RA (arrow). (**B**) Intraoperative photograph demonstrating the distal tip of the wire-like metallic fragment penetrating the right atrial wall; the penetrated tip grasped with forceps is indicated by an arrow. (**C**) Retrieved metallic fragments. The shorter fragment, indicated by an arrow, corresponds to the component that had been adherent to the surface of the RV, whereas the longer fragment corresponds to the component removed from the RA. RA, right atrium; RV, right ventricle

After snaring the superior and inferior venae cavae, the RA was opened obliquely to allow removal of the wire fragment under direct visualization (**Fig. 3A**). This atriotomy was separate from the actual perforation site in the right atrioventricular groove. Inspection of the RV cavity through the tricuspid valve revealed no intraventricular fragment. However, inspection of the RV surface revealed another small wire fragment embedded in the epicardium, which was removed (**Fig. 3B**). The retrieved fragments are shown in **Fig. 3C**. The fragment appeared to be a thin, flexible metallic wire component. Although its mechanical strength was not formally tested, the intraoperative impression was that repeated bending could readily lead to fracture. No additional fragmentation was observed during removal. This fragment showed no direct anatomical continuity with the right atrial perforation site and was therefore considered to represent a detached wire component that had floated within the pericardial space after separation from the main fragment and had subsequently adhered to the epicardial surface of the RV. After fragment removal, the right atriotomy was closed primarily, and the perforation site was closed separately with pledgeted mattress sutures.

After completion of the intracardiac procedure and successful weaning from cardiopulmonary bypass, the venous and arterial cannulas were removed, and heparin was reversed with protamine. Postoperative anticoagulation with intravenous heparin was resumed on the morning after surgery once hemostasis had been confirmed, followed by warfarin on POD 2. Heparin was continued until the PT-INR reached the therapeutic range. The postoperative course was uneventful. Follow-up contrast-enhanced CT demonstrated no recurrent pericardial effusion or IVC obstruction. Although a focal narrowing of the IVC (11 × 6 mm) was present, the lumen remained patent. A retained wire component extending from the anterior caval wall toward the vertebral body was also identified. She has remained clinically stable without recurrent pericardial effusion or IVC obstruction during 2 years of follow-up.

## DISCUSSION

The risk of IVC filter–related complications increases with longer dwell time.^1,2)^ As summarized in **Table 1**, caval wall penetration is relatively common on CT, whereas fracture, migration, cardiac perforation, and tamponade are much less frequent but may have serious or life-threatening consequences.^1–3)^ Reported incidence varies widely depending on study design, filter type, imaging modality, and implantation duration.^1–3)^ In particular, penetration rates are often derived from CT-based evaluations and include many asymptomatic cases, whereas severe complications such as cardiac perforation and tamponade are mainly described in case reports rather than large cohort studies.^4,5)^

**Table 1:** Reported incidence and major clinical characteristics of IVC filter–related complications

Complication	Reported incidence (%)	Major affected structures	Clinical relevance
Caval wall penetration	20–50	Duodenum, aorta, renal vein	Frequently detected on CT; many cases are asymptomatic
IVC thrombosis/occlusion	2–30	IVC, iliac veins	May cause lower limb edema
Filter fracture	1–16	RA, RV, pulmonary artery	Risk increases with longer dwell time
Filter migration	3–5	RA, RV, pulmonary artery	May cause arrhythmia or cardiac tamponade
Cardiac perforation/tamponade	<1	RA, RV, pericardium	Rare but potentially life-threatening

Reported incidence varies depending on study design, filter type, imaging modality, and duration of implantation. Incidence values should be interpreted as reported ranges rather than true population-based rates. Data were derived from previous reports.^1–5)^

IVC, inferior vena cava; RA, right atrium; RV, right ventricle

In the present case, a permanent filter fractured more than a decade after implantation, resulting in delayed RA perforation and cardiac tamponade. This course highlights that permanent devices are also susceptible to late structural failure and that severe complications may occur long after implantation. CAG was initially performed because of epigastric pain, ECG changes, and mild elevation of cardiac enzymes. No pericardial effusion was detected at that time, suggesting that tamponade did not occur immediately after RA perforation but evolved gradually over time. Although retrievable filters are generally associated with higher rates of penetration and fracture during prolonged implantation, permanent filters are also susceptible to late complications, including fracture, migration, and organ perforation.^2,3)^ Thus, even permanent filters may cause life-threatening cardiac complications more than a decade after implantation.

A particularly important diagnostic feature in this case was the temporal comparison of 3D CT images. CT obtained 4 years earlier demonstrated an intact filter configuration, whereas the current reconstruction revealed a clear structural defect caused by the disruption and loss of a wire component connecting the struts. This temporal comparison provided strong evidence of true filter fracture and supported the interpretation that the migrated fragments identified in the right heart originated from the fractured filter. Another notable feature of this case was the distinct mechanism of injury caused by different components. Multiple struts penetrating the duodenum remained attached to the filter body, whereas RA perforation was caused by a detached wire component that had migrated to the heart. This distinction helps explain the complex clinical presentation and the anatomical basis of the observed injuries.

This interpretation is also supported by the intraoperative findings. Intraoperatively, the filter body and struts remaining within the IVC were covered by white neointimal tissue, indicating chronic incorporation into the caval wall. In contrast, the migrated component was considered to be a disrupted wire element that had detached from the main filter structure. We speculate that this component either escaped complete neointimal incorporation before detachment or became mobile after mechanical fatigue and structural failure, thereby allowing migration to the heart.

For follow-up of long-term indwelling filters, plain radiography may be useful as an initial screening tool to detect gross migration, deformation, or fracture. However, CT is necessary when penetration beyond the caval wall, embolized fragments, or adjacent organ involvement is suspected, because it provides more detailed anatomical information. In the present case, only chest radiography was available, and the filter itself was outside the field of view. Moreover, the wire fragment within the pericardial space was difficult to identify on chest radiography. Therefore, CT was essential for accurate diagnosis and anatomical assessment.

Regarding the mechanism of delayed cardiac tamponade, the condition was unlikely to have resulted from acute rupture and was more likely caused by repeated microbleeding from the RA perforation site. Ongoing anticoagulation may have contributed to the persistence of bleeding, although a direct causal relationship cannot be definitively established. The migrated fragment may have moved in and out of the perforation with cardiac pulsation, gradually enlarging the defect and resulting in delayed bleeding into the pericardial space. The metallic fragment identified on the RV surface was not considered an intraventricular embolus, but rather a detached wire component that had separated after repeated friction against the pericardium and subsequently adhered to the RV surface. This mechanism may also explain the transient improvement in symptoms during the initial hospitalization: the protruding fragment may initially have caused symptoms through mechanical irritation of the pericardium, whereas its subsequent detachment may have temporarily alleviated these symptoms. Nevertheless, persistent microbleeding from the perforation site may have gradually led to pericardial effusion and delayed cardiac tamponade.

From a therapeutic perspective, an endovascular approach was considered unsuitable because the fractured components had penetrated multiple structures, including the duodenum, RA, and pericardium. Therefore, a sequential open surgical approach consisting of laparotomy followed by median sternotomy was chosen to permit safe removal of the fragments and definitive repair of both abdominal and cardiac injuries. This approach resulted in a favorable postoperative course without recurrent pericardial effusion or IVC obstruction. This case highlights the importance of long-term surveillance and continued awareness of late complications, even in patients with permanent filters. Filter-related complications should be considered when such patients present with atypical chest or abdominal symptoms, regardless of the time elapsed since implantation.

## CONCLUSIONS

Permanent IVC filters can fracture and cause delayed, life-threatening cardiac complications even many years after implantation. In patients with a history of IVC filter placement, atypical chest or abdominal symptoms should prompt consideration of filter-related complications and appropriate imaging evaluation.

## DECLARATIONS

### Use of artificial intelligence

During the preparation of this manuscript, the authors used generative artificial intelligence tools to assist with language editing and structural refinement of the text. The authors reviewed and edited all content and take full responsibility for the accuracy and integrity of the manuscript.

### Availability of data and materials

The datasets supporting the conclusions of this article are included within the article.
